# The Model of “Informed Refusal” for Vaccination: How to Fight against Anti-Vaccinationist Misinformation without Disregarding the Principle of Self-Determination

**DOI:** 10.3390/vaccines9020110

**Published:** 2021-02-01

**Authors:** Stefano D’Errico, Emanuela Turillazzi, Martina Zanon, Rocco Valerio Viola, Paola Frati, Vittorio Fineschi

**Affiliations:** 1 Department of Surgery, Medicine and Health, University of Trieste, 34149 Trieste, Italy; sderrico@units.it (S.D.); martina.zanon@virgilio.it (M.Z.); 2 Department of Surgical Pathology, Medical, Molecular and Critical Area, Institute of Legal Medicine, University of Pisa, 56126 Pisa, Italy; emanuela.turillazzi@unipi.it; 3 Department of Anatomical, Histological, Forensic and Orthopaedic Sciences, Sapienza University of Rome, Viale Regina Elena 336, 00161 Rome, Italy; roccovalerio.viola@uniroma1.it (R.V.V.); paola.frati@uniroma1.it (P.F.); 4 IRCCS (Istituto di Ricerca e Cura a Carattere Scientifico) Neuromed Mediterranean Neurological Institute, Via Atinense 18, 86077 Pozzilli, Italy

**Keywords:** vaccination, refusal, misinformation, anti-vaccination movement, vaccine injury compensation

## Abstract

Vaccines are arguably a public health success story as well as an incredibly cost-effective medical resource. Despite this, worldwide concerns about their safety are growing, with the risk of increased morbidity and mortality in vaccine-preventable diseases because of vaccine refusal. The global political trend in developed countries is to increasingly reduce mandates and the compulsory nature of vaccination programs. This is due to strong opposition from anti-vaccination movements and groups. While these have existed since the beginnings of vaccinology, they have recently gained a strong foothold through massive exploitation of the media and especially the internet. This has led to widespread misinformation and greater difficulty for governments and health institutions in dealing with parents’ concerns and misconceptions. Common strategies in order to maintain a high degree of public acceptance of vaccines include the enhancement of adverse effect reporting systems, the enrichment of scientific literature, and the dissemination of targeted information to parents and health care providers. Vaccine risk perception, in fact, largely exceeds the evidence and is linked to well-known general population cognitive bias, which must be recognized and corrected. Although there is no doubt about the convenience of universal vaccination, a lively international debate is underway with regard to the legitimacy of mandatory vaccination programs. Most scientists agree that the individual’s right to self-determination should be preserved. The only way to simultaneously protect the right to health is to introduce an informed refusal model, which aims to guarantee the highest coverage rates for vaccination.

## 1. Introduction

Vaccines preventing infectious diseases are counted among the greatest public health achievements [1] and are a cost-effective medical resource. A clear example is smallpox eradication, which has enabled thousands of lives and billions of dollars to be saved annually [2]. Despite this, in industrialized countries worldwide, there is a growing concern about vaccine safety, which has led to the undermining of vaccination programs. The H1N1 influenza pandemic of 2009 and 2010 revealed a strong public fear of vaccination [3], with 70 million doses unused in the United States [4] in spite of there being no evidence of harm from vaccination.

Public health authorities fear that a decline in vaccination rates may fall under the “herd immunity” threshold even for well-established campaigns, resulting in the free circulation of pathogens, multiplied risks and increased morbidity and mortality. This is already happening in the U.S., where there has been a return of nearly forgotten vaccine-preventable diseases (VPDs). From 2001 to 2008, a median of 56 (range: 37–140) measles cases were reported annually to the Centers for Disease Control and Prevention and in 2010, 9143 cases of pertussis were reported in California, the highest number reported in the last 63 years [5]. Among them were ten infants who died from the disease [6]. Measles surveillance in Europe reported 9579 cases in the 12-month period from April 2013 to March 2014: 26.5% of cases were recorded in Italy with the lowest rate of vaccinations ever reported in the last decade [7].

Anti-vaccine groups usually promote undefined concerns that immunobiologics constitutes the injection of foreign material into the body, which is thought to somehow carry additional risks. As a result of increasing pressure from these groups and resistance to compulsory vaccinations, many countries have removed cumulative penalties and introduced a conscience clause, allowing parents to refuse immunization for their children and obtain a certificate of exemption. In 1998, the government of France suspended all hepatitis B immunization programs among adolescents because of a suspected correlation with multiple sclerosis, despite reassuring advice from the World Health Organization (WHO) and other organizations [8]. When it became clear that no evidence existed to support such an association, the vaccination program was reinstated but was met with little support because of widespread, unjustified concerns [8].

In the 1990s, in the National Vaccination Program, the Italian health authorities introduced the project to gradually abolish compulsory vaccination for the years 1997–2000. A few years later, the National Vaccination Program for the years 2005–2007 indicated the following as mandatory objectives for regions: starting information campaigns about vaccine safety; setting up electronic databases; including vaccine-adverse events; achieving high vaccination rates in the population; promoting education for health practitioners. In order to legitimize this federal autonomy, the central government decided to abandon the distinction between mandatory and recommended vaccines in the national vaccination schedule for subsequent years. Several Italian regions (Veneto, Piedmont, Lombardy, Tuscany) experimentally suspended compulsory vaccination and, as a consequence, penalties were removed and a conscience clause in vaccination was introduced. The local government of Tuscany recently approved guidelines for informed vaccination.

## 2. Anti-Vaccination Movement: Learning from the Past

Misinformation in the scientific field and especially in medicine and healthcare has always been widespread. Experts from the academic environment, used to comparing data and evidence obtained through rigorous collection and analysis, are not inclined to accept the strong beliefs of those who back “pseudoscience” and “anti-science” campaigns. This attitude applies to the whole spectrum of non-scientific literature, which denies the efficacy of most conventional medicines in general, opposing not only vaccines, but also chemotherapy or preventive medicine when invasive or potentially dangerous tests are required (for instance mammography for the early detection of breast cancer). The unexpected success of campaigns of this kind can be explained by the theory of bounded rationality by Herbert A. Simon [9]. According to this model of cognitive psychology, rationality is limited in individuals making decisions as they tend most often to settle for a satisfactory solution rather than an optimal one. Of the various emotional and cognitive biases, we need to consider the special psychology of risk perception, which tends naturally to exaggerate extremely rare catastrophic occurrences and awards less importance to events, which are much more probable but less serious. On the other hand, it is possible to observe a clear trend in public opinion, which follows reassuring messages, even if unproven, rather than more reasonable ones. Another cognitive bias in risk perception is “ambiguity aversion”: i.e., when there is some uncertainty regarding the likelihood of an event deriving from an action, people deduce that the action is riskier than mean estimates [9].

Right from the start it was compulsory vaccination rather than vaccination itself which met with the strongest resistance. This explains why anti-vaccine sentiments are such long-established phenomena, being traceable to the origins of vaccinology. In 1871 during an epidemic, there was widespread objection to the Dutch government’s requirement that all school children be vaccinated [10]. Around the same time, resistance grew stronger in the United States. The Anti-Vaccination Society of America was founded in 1879, the New England Anti Compulsory Vaccination League in 1882 and the Anti Vaccination League of New York City in 1885 [11]. Even before that, in England and Wales, an anti-vaccination movement grew in reaction to laws passed between 1853 and 1871, which made the smallpox vaccination compulsory for infants (Vaccination Act 1853, 1857) with defaulting parents liable for a fine or imprisonment [12,13]. In 1889, a Royal Commission on vaccination was appointed to examine the concerns about vaccinations. After seven years of deliberations, the commission recommended the abolition of cumulative penalties, which was seen to be a compromise gesture to the anti-vaccinationists. A new Vaccination Act in 1898 introduced a conscience clause, allowing parents who did not believe vaccination was efficacious or safe to obtain a certificate of exemption, thus establishing the figure of conscientious objector [14].

The modern history of the U.S. anti-vaccination movement dates to 1982, when a TV-program entitled “DTP: Vaccine Roulette” aired on the network WRC-TV. The speakers accused the diphtheria, tetanus, and pertussis vaccine (DTP), particularly the pertussis component, of causing severe neurologic sequelae [15]. In response to this TV show, many parents refused to vaccinate their children, not only in the U.S. but around the world.

It was in 1998 in the U.K. that the most influential milestone in the development of the modern anti-vaccination movement was reached. An article by Dr. A. Wakefield suggesting a correlation between the measles, mumps, and rubella (MMR) vaccine and autism appeared in “The Lancet” [16]. Immediately, hundreds of epidemiological and biological studies denied any evidence of causal relationship and were reviewed in 2004 [17]. Furthermore, in January 2010, the British General Council issued the results of its enquiry into Andrew Wakefield’s research, concluding that he acted unethically and with “callous disregard” for his patients; consequently, he was struck off the U.K. medical register [16]. Finally, in February 2010 The Lancet formally retracted the study [18].

Nevertheless, it is well documented how the resonance in the media following the publication of Wakefield’s assumptions caused an evident decline in measles vaccination rates [19]. The belief that the MMR vaccine causes autism is still one of the most important reasons for refusing vaccination [20]. In the U.K., the detrimental impact of the spread of such erroneous suspicions caused a drop from 92% in 1995 to 84% in 2002, and probably to less than 60% in some parts of the country [21].

Similarly, vaccination coverage in other European countries such as Germany, Austria and Spain is influenced by well-publicized but unsubstantiated links between vaccines and diseases like multiple sclerosis, sudden death, and epilepsy, as well as autism [22]. A direct correlation has also been demonstrated between Wakefield’s publication and measles outbreaks, especially in some areas of the U.K. where the study met with great interest from the public [23]. This has resulted in unnecessary fatal cases and enormous additional costs because of the well-known morbidity of the disease, especially among the adult population. This may be considered a clear confutation of one of the most common theories of anti-vaccinationism, which is to deny efficacy of vaccines in fighting and eradicating infectious diseases while arguing a chronological bias due to improvement in environmental hygiene and economic conditions.

## 3. Internet and Misinformation

At present, the internet seems to be the most influential medium with regard to parents’ beliefs about immunizations. According to recent surveys, around 15% of the people interviewed searched online for information on immunizations or vaccinations. Over half (52%) of users believe “almost all” or “most” information on health sites is credible [24]. The same study observed that parents who exempt children from vaccination are more likely to have obtained information from the internet than parents who have their children vaccinated. This suggests that the availability of inaccurate and deceptive information online plays an important role.

A Swedish study of parents who postponed or abstained from vaccinating their children found that the main source of information for over 80% of respondents was the media [25]. A case–control study performed in the U.S. comparing parents of fully-vaccinated and exempt children, reported as common reasons for not vaccinating: fears that vaccines might cause harm or overload the immune system; believing their child was not at risk for the disease or that the disease was not dangerous; that it was better to develop immunity naturally rather than from vaccines or that the vaccines might not work [26]. These are all popular assertions on anti-vaccination websites. At the current time, we were able to identify well over 300 anti-vaccine internet sites from a single simple search [27]. Arguments proffered on anti-vaccination websites are analyzed in the literature to determine the extent of misinformation and to examine discourses used to support vaccination objections. The most common arguments concern the safety of vaccines, the promotion of treatments superior to vaccination (e.g., homeopathy), defense of civil liberties, fear of pharmaceutical and government conspiracies, and morality and religion [24]. An analysis of YouTube immunization videos found that 32% opposed vaccination, and that these had higher ratings and more views than pro-vaccine videos [28].

The anti-vaccination movement usually denigrates scientific studies and the scientific method in general, which is largely typical of “pseudoscience”. At the same time, it aspires towards a sort of scientific legitimacy for its theories. Its exponents present themselves as legitimate authorities with recognized credibility and institutional credentials at national or international level. Obviously, such pretensions fail to provide solid references: the majority of sites report data from many self-published works, sometimes referring to the alternative medicine press, lacking both rigorous methodological parameters and a strict peer reviewing system. Often anti-vaccination claims are presented without cited sources [29]. When research published in indexed medical journals is quoted, the conclusions drawn prove to be inconsistent with those of the authors [29]. Overall, this signifies the existence of masses of data on the dangers of vaccination. It is probably true that the internet offers the greatest risk for the public to make misinformed decisions about vaccinations without scientific knowledge and based on misleading information [30,31].

## 4. Promoting Vaccination through Information

During the last decade, Italian politics decided that a mandatory regime for vaccination could not fit any longer with the health care model shifting from medical paternalism to the therapeutic alliance. However, it still remains mandatory for the government to guarantee a proper vaccination rate in the population in order to defend the human right to health, especially for people who are clinically contraindicated for vaccination. Consequently, the only way to reach the minimum rate of vaccine uptake is to implement something like an informed consent procedure. In case of vaccination, parents, and especially mothers, are the first target as far as all the information about vaccine diffusion is concerned [32,33,34].

Nowadays, medicine and health care are dominated by the systematic search for evidence to support common practices; the best management of funds, costs and efficiency are becoming even more important in the health system governance, in order to face the enormous growth of costs in the medical field. For these reasons, even public campaigns, social advertising and health promotion interventions have been reviewed in terms of respective costs/effectiveness balance. Actually, at the moment, the data collected by different authors about this issue are not sufficiently univocal and reassuring.

On one hand, we could say that under-immunization is linked with vaccine safety concerns among parents [35]: authors observed that it was the main or the only reason for vaccine refusal in the US. On the other hand, effectiveness of corrective information about vaccine safety in modifying and orienting parents’ behavior towards vaccination has been tested with uncertain outcomes. Hendrix et al. obtained increased vaccine intentions in parents when the child’s benefits, more than the benefits of all the community, were emphasized [36]. Conversely, Nhyan and colleagues found that denying claims of links between MMR vaccination and autism led to a reduced misperception about the specific theme but did not increase parental intent to vaccination [37]. This could mean that evidence-based scientific contents may not be enough to refute incorrect beliefs [38].

Other specific communication strategies have been tested to define the most effective messages and information. Some authors focused on illustrating scientific information about risks coming from under-immunization, more exactly with warnings in the form of graphic pictures and anecdotes pointing out the severity of prevented diseases and, consequently, the dangers of failing to vaccinate [39]. This sounded like replacing wrong elements with new information, rather than simply refuting incorrect beliefs. Although this communication strategy resembles psychological terrorism, it has been proven that similar strategies are effective in other campaigns of health promotion, for instance against smoking cigarettes and driving cars under the influence of alcohol [32].

The scientific societies recognize the importance of the relational dimension in the communication from physician to patient, especially concerning vaccine acceptance [40]. 

In particular the American Academy of Pediatrics (AAP) faced the issue by a practical approach. The AAP provided clinicians with explicit advice for facing parents’ vaccination refusal, including: listening carefully and respectfully to their concerns; attempting to correct possible misperceptions and misinformation, but admitting the limitations of vaccines, even minimal; discussing each vaccine separately as concerns may be related to one or two specific vaccines; exploring the possibility that cost is a reason for refusing [41].

Dealing with the improvement of vaccination among the targeted population in general, in the specialized literature we can find some proposals aimed to enhance the coverage rate through a wide range of extended mass information. In a concise and pragmatic editorial [42], Poland et al. proposed a synthetic program which consisted of: keep studying and publishing simultaneously about real vaccine safety through monitoring programs, and about general public concerns; making compensation available to anyone; enhancing public education and persuasion by introducing specific training for health care professionals in countering anti-vaccinationists’ false claims.

We agree with this advice and firmly recommend that immunization campaigns should be designed to target society in general. To do so, a few key principles should always be taken into account. First of all, it is of paramount importance that any immunization programs have a communication strategy integrated into the planning from inception [43]. Information should be provided according to a precise algorithm in which different facilities and subjects are well recognizable; a structured hierarchy should be established to define each kind of communication to promote for any level of the chain. The top level is obviously represented by the head of the Health Ministry and the National Government, while at the bottom, there is the widespread network of local health authorities, general practitioners and pediatricians. In the middle, other institutions and civil organizations, those interested in health promotion, should be involved in the general program so that contents and manner of communication are shared. In this complex system, physicians’ organizations would also have a precise role from an institutional perspective, exercising disciplinary proceedings if necessary.

A coordinated advertising campaign can reveal itself to be more effective in promoting a definite message through empowerment and amplification of its meaning due to the coherence of different levels of information. Similarly, the communication strategy must implement all the media technologies with the proper formats, combining different ones and preserving at the same time the perception of a unique project. Moreover, the environment to be focused on is always the school, considering that, according to a recent meta-analysis about vaccination programs, information disseminated by schools was more important than information provided by other media [44]. In other words, it is fundamental to plan in detail the communication because advertisements or isolated interventions may be unproductive while tailored campaigns are more likely to meet the needs of communities.

Nonetheless, when we discuss the most suitable solution in promoting vaccination, we should remember that a few parents, already deeply convinced about risks and ineffectiveness of vaccination, will not change their mind on a rational or scientific basis. These people represent the radical anti-vaccine movement followers and cannot be targeted by any pro-vaccine campaign; maybe they would resist even legal requirements. The only strategy should be to keep them isolated (in a figurative sense) and target sceptic people immediately around them who could be doubtful about advantages in vaccination; in this kind of situation the AAP gives some tips to providers to face parental skepticism, especially they recommend leaving the door open to a further re-evaluation of the issue in the near future.

We have already talked about common misperception and cognitive bias in the first paragraphs of this paper. One of the hardest issues to deal with is to shed light on the risk for health when it is quite delayed across human lifespan. We observe this barrier in medical information typically with Human Papilloma Virus (HPV) vaccination as girls and especially their mothers do not realize properly to what extent they are exposed to the risk of HPV-related cancers during their lives: the problem is the long lag time (even a few decades) between the specific infection, which the immunization is designed to prevent, and the onset of malignancies.

The misperceived message is that young girls (and boys) undergoing vaccination are taking all the real and present risks (that are, in fact, minimal) in the hope of a benefit, even if uncertain, at some undefined time in the future. An awareness-raising communication strategy, for example, could be to emphasize the lengthening of life expectancy to increase the perceived danger of contracting a serious cancer disease. Hence, in our society, people tend to have children at an older age than in the recent past and this means that women in their 50s are still likely to have young boys or girls to take care of. Young people may be more interested in aspects of desirable quality of life than in mere longevity. Then, they could hope to be strong enough to keep practicing their common activities to realize their self-identities. All these arguments should clarify the importance of lowering as much as possible the risk of falling ill because of a disease with such a high mortality and morbidity. In this sense, it would be useful to stress particularly the consequences of a diagnosis, the painfulness of the disease, the heaviness of therapies, and the effects of surgery. As we have already said, some might consider this a detrimental brainwashing approach but it is part of a fair exchange of information.

During the relational approach of medical care, it is very important to engage the whole family in this kind of decision making to promote the highest grade of awareness in each member. Although physicians may be scared about losing a part of their authority and professional autonomy [45], they should accept what is emerging as a new family-centered model in health assistance. In this way, it is easier to make people understand that they must take all the necessary decisions as early as possible, to safeguard their current and their future health. As a further step, it would be desirable that people perceive vaccination as a social norm, part of their civil duties and be proud of their choice. With all this in mind, they should be aware that they are participating in a common process of disease eradication, even if they may not live long enough to see the achieved goal, because usually it takes more than a lifespan.

## 5. Vaccines Safety: Evidence beyond Misperception

As reported, increasing public mistrust of vaccination is caused mainly by concerns about vaccine safety. We have already mentioned cognitive bias. In the case of vaccine risk misperception, people are influenced by at least three cognitive determinants: the desire to find order and predictability in random data; a difficulty in detecting and correcting biases in incomplete and unrepresentative data; an eagerness to interpret ambiguous and inconsistent data to fit theories and expectations [46]. This is much more structured and erroneous than a simple “confirmation bias” [47]. Furthermore, misinformation typically generalizes the risk of adverse reactions for all vaccination, while we should consider those related to each vaccine as well as the age of each patient. It is recognized, for instance, that awareness of the association between the smallpox vaccine and encephalopathy and encephalitis has led to the incautious attribution of severe events, such as sudden infant death syndrome (SIDS), epilepsy and chronic neurological disability to the DPT vaccine [48]. It is thus clear that vaccine-related adverse reactions must be monitored and weighed carefully before establishing causal relations [49]; even a clear correlation, despite extremely rare events, will reveal itself to be weak when submitted to extensive meta-analysis and systematic reviews [50,51].

Since 2012, the WHO has included observed rates of adverse reactions in information sheets available on a dedicated institutional website [52]. By now, most evidence correlates mild adverse reactions with vaccines that are currently distributed and recommended or mandatory in developed countries. These include fever, cough, local pain due to the injection, irritability, drowsiness, vomiting and similar. Anaphylactic events are reported but they are extremely rare. In the U.S.A., the Institute of Medicine of the National Academies of Science has been reviewing literature regarding the adverse effects of vaccines since 1986 on behalf of Congress [53]. The latest reviews conclude that the most rare and severe adverse effects, like encephalitis, epilepsy and developmental disorders, have been consistently reduced through drug surveillance and medical exemptions to vaccination, mainly due to immunodeficiency [54].

All developed countries have been improving their adverse drug reaction reporting systems over recent decades with special attention given to vaccines [55]. There is no doubt that active immunization deserves special attention in every country, regardless of if it is a national requirement, because it is administered to a healthy part of the population, mainly newborn and children, and it may have adverse effects, even though benefits largely outweigh risks. This is the reason why a fair no-fault compensation scheme should be maintained in all these countries.

## 6. Vaccine Injury Compensation

Vaccine injury compensation programs have been in use since the 1960s, the first being introduced in Germany in 1961. This was soon followed by France and other European countries while the U.S. followed suit only in 1986. Nowadays at least 19 countries have a program, including Canada although only by a federal law in the state of Quebec [56].

Schemes throughout the world vary in structure and approach. Common components include: administration (national, federal, other); eligibility and vaccines covered; types of compensation; litigation rights [56]. Usually they are supported directly by governments or, in a few exceptions, by pharmaceutical insurance, as in the cases of Sweden and Finland. In the U.S.A. costs are covered by a special excise paid by purchasers, at an average amount of 0.75 dollars per dose [57]. They are designed to cover the highest number of events possible, requiring only a general causal allegation rather than etiological proof of the specific case, extending compensation on the basis of presumption. This is formally established only in the U.S.A., where there is a vaccine-injury table, so that if there is a specific adverse reaction to a definite vaccine in the allowed time interval, a “legal presumption of causation” is provided to the claimant.

Nevertheless, it seems that most of the other schemes, even the French one, which in theory requires clear and convincing evidence, allow a degree of flexibility in favor of the injured person. The common basic policy purpose, in fact, is to divert claims for vaccine injury away from civil courts, providing a better opportunity to deal with the standard of proof required. In fact, the implementation of a no-fault compensation paradigm avoids the typical delays and uncertainties of the tort litigation system [58], at the same time offering the advantage of a reasonable liability protection for healthcare providers and vaccine manufacturers. In Italy, Law No. 210 has, since 1992, established economic indemnity in favor of persons who are permanently impaired after compulsory vaccinations. This system is defined within the framework of social security that should guarantee partial indemnity to the victims of damaging episodes unrelated to any medical malpractice or third-party responsibility. The principle is founded on social solidarity and the burden of the consequences of the damage is converted to collective responsibility [58]. 

Regardless of these technical discrepancies, such schemes are more likely to function if they are part of a well-established, comprehensive national social welfare system.

It has not yet been demonstrated whether the introduction of such programs improves vaccine uptake. Ironically, the better they work and the more they compensate claims, the more likely it is that risk perception may increase, thus dissuading parents from vaccination [59]. Nevertheless, we agree that such an integration of modern welfare systems sits well with basic considerations of justice and equity [60]. The availability of a no-fault compensation scheme for immunization, unlike other medical procedures, comes from the awareness that it provides benefits to the whole society and an individual’s participation should not be only a question of free choice. Moreover, its existence may indirectly improve adverse reaction surveillance, eventually helping in the identification of predictable sources of injury and vulnerable subpopulations [61].

## 7. The Ethical Issue of Mandatory Vaccination

Vaccines have proven their efficacy mainly through compulsory uptake; as we reported above, the faculty of refusal was obtained quite early in the U.K. and the U.S.A. due to strong opposition from the anti-vaccination movement. A deeply rooted Protestant heritage on one side and strong liberal identity on the other placed at the center the individual’s rights and claim to self-determination. In any case, most people have continued to trust government recommendations, and vaccine uptake rates are nowadays sufficiently high in most cases.

During the last few years, the issue of mandatory vaccination legitimacy has reached European countries which, like Italy and France, have a totally different cultural background. At the same time, scientists and clinicians participate in the global debate about the opportunity to reduce legal vaccine exemptions or to make them harder to obtain [62] because there is concern about vaccine-preventable diseases returning [63,64]. Kennedy et al. conducted a national survey in the U.S.A. to investigate parents’ beliefs in refusing vaccination. After comparing type (medical, philosophical, religious) of exemption allowed by States as well as ease of obtaining such exemption, they hypothesized that laziness could be an important factor in claiming exemptions [65]. Obviously, the adoption of time-consuming procedures for refusal to promote vaccination is an effective strategy but it does not deal with the ethical issues involved.

Authors requesting a mandatory regime consider public health to be paramount and that the community should have the right to protect itself from bad health decisions [66]. They also support the question of justice in minimizing “free-riders” [67], i.e., those who benefit from herd immunity without facing the risk of vaccination themselves.

On the other hand, although vaccination safety and efficacy are evident, much of the literature supports the ethical and philosophical unfairness of mandatory uptake as it contrasts with individual autonomy and freedom of choice in health matters [68,69]. Furthermore, it is claimed that compulsion is unnecessary, and that evidence of its usefulness is unconvincing [70]. In Europe, for instance, there seems to be no significant difference in population coverage between countries only recommending and those obliging the same vaccinations [71].

Others reject a purely ethical approach and maintain a pragmatic position, affirming that a mandate should be instituted if it is the only way [72]. Compulsoriness might be abandoned when high coverage has been achieved through other efforts, because it could be counterproductive, causing a public backlash if strongly convinced parents are forced to comply [73]. Similarly, it would be reasonable to balance political decisions with the specific historical, social and epidemiological context [74].

## 8. Conclusions

The efforts of anti-vaccinationists have had disruptive and costly effects, including damage to individual and community wellbeing through outbreaks of previously controlled diseases, the withdrawal of vaccine manufacturers from the market, the compromising of national security (in the case of anthrax and smallpox vaccines), and lost productivity [42].

The nature of the internet allows all opinions to spread widely and instantaneously; it seems that an inadequate scientific knowledge base within the media combined with the irresponsible tendency to sensationalize has helped anti-vaccination groups to instill fears and concerns among the general public [27]. What solutions exist to quell such unjustified fears? Is immunization against misinformation possible?

The publication of high-quality studies to investigate the sociodemographic characteristics of the “conscientious objector” may not be sufficient [75,76]. Above all, healthcare professionals, parents and patients must be correctly informed to counter the false and injurious claims of anti-vaccinationists [77]. We should not ignore the effect that spreading misconceptions about vaccination risks has had on health providers [78]; neither should we assume that parents who currently immunize will continue to do so [79]. Patients and parents seek to balance risks and benefits. This process must start with increasing scientific literacy at all levels of education. If trust in government and public health is maintained, a free society will benefit more in a long-term perspective from an immunization program based on promotion rather than prescription.

The role of the government is to inform, educate, recommend, and even provide incentives for immunization but not to require acceptance without exclusion from the civilian population.

A normative model for “informed refusal” comes from Latvia where healthcare providers are obliged to obtain written and signed decline statements from whoever refuses to vaccinate, only after all health consequences have been fully explained to no avail [80]. This must remain a reasonable choice in a free democracy with a culture of informed consent, as it allows both religious and philosophical exemption.

Universal coverage of proven effective vaccines is still undeniably a principal measure for maintaining and improving public health. It would be an unforgivable error to allow the vaccination rates to decline below the critical percentages of herd immunity; it is probable that if this happens, VPD outbreaks will be serious enough to force governments to re-establish compulsoriness as in this case it would seem ethically justifiable [81]. This would constitute a double failure, considering on the one hand the morbidity and mortality that could otherwise be prevented and on the other the legal disregard of the individual’s self-determination and freedom of choice [82]. On the contrary, the achievement of the programmed vaccination coverage following a non-compulsory administration would represent a win–win situation for the health of the community and the right to self-determination of each individual.

## Data Availability

Not applicable.
